# Coral Dermatitis or Infectious Dermatitis: Report of a Case of Staphylococcus Aureus Infection of Skin After Scuba Diving

**DOI:** 10.7759/cureus.2196

**Published:** 2018-02-16

**Authors:** Venkataramana Kandi

**Affiliations:** 1 Department of Microbiology, Prathima Institute of Medical Sciences

**Keywords:** skin lesion, coral dermatitis, staphylococcus aureus, dermatitis, staphylococcal skin infection

## Abstract

Skin lesion which develops after deep sea diving is termed as coral dermatitis. The corals are known to produce a toxic substance which when comes in to contact with human skin may elicit hypersensitive reactions. Most previous reports highlight the allergic reactions caused by deep sea diving. This is a rare case of staphylococcal skin infection in a second-year medical student caused by *Staphylococcus aureus*; he reported a history of deep sea diving before being presented to the hospital with skin rashes. This case highlights the importance of considering infectious aetiology in cases of coral dermatitis.

## Introduction

Coral dermatitis is a skin condition caused by corals. These are a group of invertebrate and immobile living organisms that belong to the kingdom *Animalia*, phylum *Cnidaria*, and class *Anthozoa*, order *Actniaria*. They include the sea anemones, sea pens, jelly fish, and the hydra [1]. Corals grow as colonies containing polyps (sac-like structures) measuring few centimetres in length. They secrete calcium carbonate and over a period, develop hard skeleton-like structures under the oceans. Corals live by capturing small fishes, see weeds, microscopic zooplanktons and derive energy from the unicellular dinoflagellates (algae) which live/grow on them. The polyps of the corals contain tentacles/organelles named as nematocysts which produces a toxic substance. It is known to cause both acute and delayed type hypersensitive/allergic reaction, resulting in skin lesions in humans, very similar to contact dermatitis. Reports of allergic skin conditions caused by various species of corals including *Phyllodiscus semoni* (night anemone), *Alicia mirabilis* (berried anemone), *Anemonia viridis* (snake locks anemone), *Haloclava producta *(ghost anemone), and *Bartholomea annulate* (corkscrew anemone/ringed), are available in the literature [2]. The skin lesions present as urticarial or vesiculo-bullous plaques, firm, and localised papules, immediately or after hours of exposure to the corals. In some people, superficial epithelioid granulomas were also noted [3].

*Staphylococci *are a group of gram-positive cocci, which form clusters. They are associated with several human infections, which include superficial skin infections to deep-seated and invasive infections. *Staphylococci *can be present as a normal flora of human skin and mucus membrane and might invade through the skin during an injury [4]. They also can develop multiple drug resistance, as evidenced by the strains called as methicillin-resistant *Staphylococcus aureus* (MRSA), and vancomycin-resistant *Staphylococcus aureus. *Infection with such strains could lead to increased morbidity [5]. Immunocompromised individuals, young children, geriatric age people and those who are debilitated with other disorders might suffer from invasive infections of *Staphylococci *[6].

Considering the potential of *Staphylococci*, it becomes important to clinically suspect, diagnose, and appropriately manage patients infected with it to reduce the complications. This is a rare case report of staphylococcal skin infection following deep sea diving or scuba diving.

## Case presentation

A 20-year-old young male medical student presented to the dermatological outpatient department (OPD) with complaints of erythematous skin rash spread throughout the body. The rashes were observed on the hands, legs, and on the trunk. The patient gave a history of scuba diving three days before he started to notice these skin lesions. The patient was found to be very anxious but otherwise was clinically normal. The patient was referred to the department of clinical microbiology for further microbiological evaluation. The skin rash was isolated but present in many areas of the body including the hands and legs. Most skin lesions coincided with the cuts. The palms and soles showed no signs of rash. There were multiple areas of erythematous skin, with raised and white fluid filled bumps. The area surrounding the affected skin was thoroughly cleansed with 70% alcohol. A sterile scalpel blade was then used to scrape the affected area. The sample was then inoculated into a blood agar, and nutrient agar and incubated at 37^0^ C.

**Figure 1:**
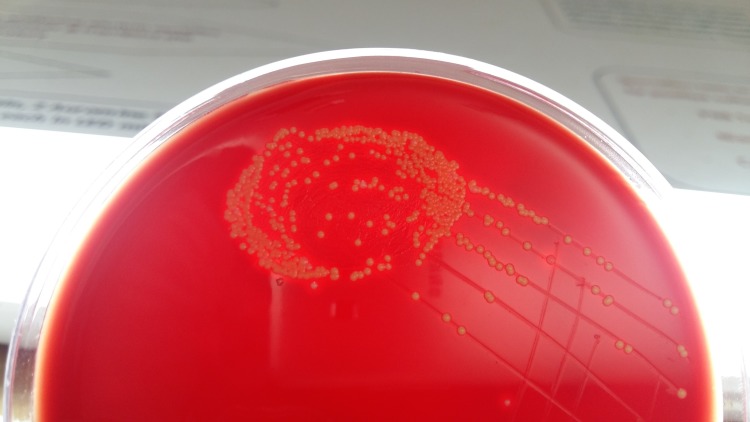
Yellow pigmented opaque beta-haemolytic colonies of Staphylococcus aureus on blood agar

The sample was also smeared on a clean and grease free slide. On the grams stain, occasional gram-positive cocci in clusters were seen. Microscopy (KOH mount) for fungal elements was negative. After overnight incubation, a moderate growth of 2-3 mm small, round, moist, convex, and opaque beta-hemolytic colonies were observed as shown in Figure 1.

 

The colonies showed yellow coloured non-diffusible pigment on the nutrient agar. Grams stain from the culture confirmed the presence of gram-positive cocci in clusters. The cultured bacterium was confirmed as *Staphylococcus aureus* by a positive coagulase test, and fermentation of mannitol. Antimicrobial susceptibility by Kirby-Bauer disk diffusion was performed, and the results showed that the isolated bacterium was sensitive to penicillin, oxacillin, erythromycin, vancomycin, linezolid, amikacin, gentamicin, ciprofloxacin, ofloxacin, levofloxacin, azithromycin, trimethoprim-sulfamethoxazole, cefepime, ceftriaxone, ceftazidime, piperacillin-tazobactam, and resistant to only cloxacillin. Since the isolated bacterium was sensitive to most antimicrobial agents tested, and was not a methicillin-resistant *Staphylococcus aureus *(MRSA) strain, and the patient was immunocompetent with no co-morbidities, and there were no signs of systemic spread, a decision to use topical antibiotic was taken. The patient was advised topical mupirocin (Bactroban®), over the skin lesions, which healed completely after 7-10 days.

## Discussion

There are several reports of coral dermatitis in the literature. Most cases have been attributed to the hypersensitive reaction elicited by the immune system against toxic substances produced by the corals. Clinically, coral dermatitis can be acute, delayed, and chronic depending on the duration of appearance of skin lesions [7]. They can present as acute urticaria, acute vesiculobullous dermatitis, subacute fleshy granulomatous dermatitis, and chronic lichenoid dermatitis.

There are only a few reports in the literature that have associated the occurrence of microbial infections in patients with allergic conditions and with a history of deep sea diving or sea bathing. A recent study by Inada et al. had observed that infection by* Kocuria* *koreensis* had complicated the condition in a patient who was already suffering from atopic keratoconjunctivitis [8].

Another report by Chen et al. had noted that patients with atopic dermatitis might be predisposed to *Staphylococcus* *aureus* and a *Herpes simplex* virus infection [9].

The literature search for bacterial skin infection after deep sea diving/sea bathing revealed only one such infection by *Staphylococci* causing folliculitis in a patient [10].

In the present case, the bacteria colonized on the skin could have been responsible for the skin lesions. The isolated bacterium was sensitive to most antibiotics and was a methicillin-sensitive *Staphylococcus aureus*. Since the patient did not have any underlying predisposing factors that could lead to systemic spread/infections, the lesions healed after the treatment with topical antimicrobial agents.

## Conclusions

Although it is an established fact that the venomous/toxic substance secreted by the corals result in some hypersensitive skin reactions in humans, it is important for dermatologists to also rule out the infectious causes. In the era of prevalence and spread of microorganisms resistant to several antimicrobial agents, timely identification and initiation of appropriate antibiotic therapy would certainly reduce the morbidity and improve patient care.
